# A comprehensive analysis of vasculogenic mimicry related genes to predict the survival rate of HCC and its influence on the tumor microenvironment

**DOI:** 10.3389/fgene.2024.1437715

**Published:** 2024-12-19

**Authors:** Jingyun Wang, Rong Gao, Jian Qi, Yingru Xing, Bo Hong, Hongzhi Wang, Jinfu Nie

**Affiliations:** ^1^ School of Medicine, Anhui University of Science and Technology, Huainan, China; ^2^ School of Basic Medical Sciences, Anhui Medical University, Hefei, Anhui, China; ^3^ Institute of Health and Medical Technology, Hefei Institutes of Physical Science, Chinese Academy of Sciences, Hefei, China; ^4^ Department of Clinical Laboratory, Anhui Zhongke Gengjiu Hospital, Hefei, China; ^5^ Hefei Cancer Hospital of CAS, Hefei, China

**Keywords:** hepatocellular carcinoma, vasculogenic mimicry, molecular pathology, biological information, tumor microenvironment

## Abstract

**Objectives:**

Investigate the predictive value of Vasculogenic mimicry (VM) related genes for the survival and prognosis of Hepatocellular carcinoma (HCC) patients and its role in the tumor microenvironment (TME).

**Methods:**

VM-related genes were obtained from previous literature, the expression profiles, single-cell data and clinical information of HCC patients were downloaded from public databases. The HCC patients were divided into different clusters by unsupervised clustering, the differences in prognosis and immune characteristics of VM-related clusters were analyzed. A prognostic model related to VM (VM Score) was constructed based on LASSO regression and univariate and multivariate Cox regression, the correlation between this model and chemotherapy drugs and immunotherapy was studied. Seurat package was used to standardize single-cell data for single-cell level analysis. The expression of risk factors in VM Score was verified by RT-qPCR.

**Results:**

VM Score composed of SPP1, ADAMTS5 and ZBP1 was constructed and validated. VM Score was an independent prognostic factor for HCC. Through the analysis of single cell data further reveals the VM Score influence on TME. In addition, VM Score could provide ideas for the selection of immunotherapy and chemotherapy drugs. RT-qPCR showed that the expression of risk factors was different in HCC cell lines.

**Conclusion:**

Our results suggest that VM Score may serve as a promising prognostic biomarker for HCC and provide new ideas for immunotherapy in HCC patients.

## 1 Introduction

Liver cancer is one of the challenges threatening human health globally, and the annual incidence of liver cancer is expected to exceed one million cases by 2025, with hepatocellular carcinoma (HCC) being the most common type of liver cancer, accounting for about 90% of all liver cancer cases (Llovet et al., 2021). Meanwhile, hepatocellular carcinoma is one of the top five causes of cancer deaths in the Chinese population (Chen et al., 2016). Given the increasing incidence and mortality of hepatocellular carcinoma worldwide, the development of new biomarkers to better facilitate therapeutic interventions has become a hot topic of research.

It is now widely accepted that solid tumors require an adequate blood supply for growth. When solid tumors grow to a certain extent, new blood vessels need to be formed to maintain an adequate blood supply to avoid tumor necrosis due to ischemia (Liu et al., 2023).In 1999, Maniotis et al. first proposed the concept of angiogenic mimicry (VM) (Maniotis et al., 1999), which is an epithelium-independent mode of microcirculation of tumors that degrades the basement membrane and extracellular matrix by providing blood perfusion and promoting the secretion of protein hydrolases by tumor cells (Wagenblast et al., 2015).VM is considered a model for neovascularization in aggressive tumors (Andonegui-Elguera et al., 2020). VM has been observed in a variety of human malignancies and is strongly associated with tumor proliferation, metastasis, and poor patient prognosis (Kirschmann et al., 2012; Hendrix et al., 2016; Tang et al., 2016; Qu et al., 2017).

Tumor microenvironment (TME) refers to the internal and external environments during tumorigenesis, growth and metastasis. The tumor microenvironment consists of immune cells, mesenchymal stromal cells, and various cytokines (Hanahan and Coussens, 2012). The tumor microenvironment utilizes benign and malignant cells to promote the harsh, immunosuppressive, and nutrient-poor environment necessary for tumor cell growth, proliferation, and phenotypic flexibility and variation, as well as the composition of the TME significantly influences resistance to malignant tumor therapy (Khalaf et al., 2021); for example, cancer-associated fibroblasts (CAF), which are one of the most abundant mesenchymal cells, one of the most abundant cells in the tumor microenvironment, involved in drug resistance to cancer therapeutics (Mao et al., 2021). Immune imbalance in the tumor microenvironment is an important feature of cancer (Binnewies et al., 2018), however, the TME of hepatocellular carcinoma in the context of VM features remains unclear. Therefore, it is essential to understand the correlation between VM and hepatocellular carcinoma.

In this study, we constructed a scoring model (VM Score) associated with VM, which included three risk factors, SPP1, ADAMTS5, and ZBP1, revealing potential prognostic biomarkers for hepatocellular carcinoma. The column line graphs created based on the VM Score were clinically informative. The association between the VM Score and immune checkpoint molecules and chemotherapeutic agents may provide new ideas for the clinical application of immunotherapy and chemotherapeutic agents. In addition, the analysis of single-cell data allows for a deeper exploration of the major cell types and cell communication roles of VM-related genes affecting TME. In conclusion, the results of this study will provide new insights into the effects of VM-related genes on hepatocellular carcinoma and validate that the VM signature can be used as a novel biomarker for hepatocellular carcinoma, which will help to improve the efficacy of individualized treatment for hepatocellular carcinoma patients.

## 2 Materials and methods

### 2.1 Data collection and preprocessing

The role of VM in cancer was explored based on literature reports and the collected VM-related genes were intersected with hepatocellular carcinoma transcriptome data to obtain a total of 18 genes for subsequent analysis (Luo et al., 2020).Hepatocellular carcinoma datasets were obtained from The Cancer Genome Atlas (TCGA) and Genotype-Tissue Expression (GTEx), and the data were downloaded from UCSC Xena (http://xena.ucsc.edu/).371 samples from TCGA-LIHC containing gene expression and clinical information were used as a training set, and GTEx (n = 110) was used to compare the tumor and normal samples genetic changes between tumor and normal samples. In addition, the GSE14520 and GSE149614 datasets from the Gene Expression Omnibus database (GEO, https://www.ncbi.nlm.nih.gov/geo/) were included in the study. GSE14520 (n = 488) was an independent external validation set and GSE149614 (n = 18) was used for single-cell analysis. Data preprocessing was performed using R software version 4.3.1 (https://www.r-project.org/).

### 2.2 Analyzing VM subtypes based on unsupervised clustering

To identify different molecular clusters based on the expression of VM-related genes, unsupervised clustering analysis was performed on each sample of the dataset using the ConsensusClusterPlus package, the number of clusters was set to two to six, and 1,000 repetitions were carried out to ensure the stability of the classification, and the optimal clusters were calculated based on the consensus matrix and the consensus cumulative distribution function (CDF). Principal Component Analysis (PCA) dimensionality reduction was used to demonstrate the distributional differences between different clusters. Kaplan-Meier (KM) survival analysis and log-rank test were used to analyze the prognostic value of subtypes. Relationships with other clinical variables were visualized using the ggalluvial package, and *p* ≤ 0.05 was considered statistically significant.

### 2.3 Functional enrichment analysis of different isoforms

The limma package was used to adopt |logFC| > 0.5 and adj. P.Val <0.05 as the thresholds for screening differential genes, the clusterProfiler and enrichplot packages were used for KEGG (Kyoto Encyclopedia of Genes and Genomes) and GO (Gene Ontology) analysis, and the results were visualized using the ggplot2 package.

### 2.4 Immune score and TMB score

To explore the effect of this gene signature on TME in HCC patients, the ssGSEA algorithm was used to calculate the degree of infiltration of different VM clusters in 28 immune cells and the epic score was used to assess differences in the tumor microenvironment between different subgroups of patients. Using somatic mutation information, TMB scores were calculated for each sample excluding exon lengths (38 million) and using median scores as cut-off values to categorize different subtypes into high and low TMB groups.

### 2.5 Development and validation of prognostic models

Genes associated with prognosis in VM subtypes were extracted by univariate Cox regression analysis (*p* < 0.05) using the survival package. Further screening was performed using the Least Absolute Shrinkage and Selection Operator (LASSO) algorithm and multivariate Cox regression analysis, and the expression of the genes and their weights would be used in the construction of the risk score model according to the following formula:

Risk Score = $\sum_{i}expi$ ∗ βi (expi is the expression level of the gene, βi is the coefficient of the selected gene in the multivariate COX analysis).

The samples will be categorized into high and low-risk groups based on the median risk score and the survival package will be used to compare the difference in prognosis between the high and low-risk groups, and the timeROC package will be used to calculate the area under the curve (AUC) to assess the accuracy of the prognostic model.

The relationship between VM Score and clinical characteristics was analyzed using multivariate Cox regression. We calculated the risk ratios of risk scores to clinical traits using the multivariate Cox algorithm, screened for independent risk factors, and then created column-line plots using the nomogram function of the rms package, which included risk scores and age. We also plotted calibration curves and decision analysis curves (DCA) to validate the difference between our model and actual observed patient survival.

### 2.6 Single-cell sequencing data analysis

Single-cell analysis was performed using the GSE149614 dataset. The raw data consisted of 31,286 cells from 18 patients. First, quality control and batch correction were performed using Seurat and Harmony packages. Cells with nCount_RNA < 4,000, nFeature_RNA < 500, or mitochondrial genes more than 15% were filtered out, and the first 15 principal components after PCA analysis were retained for tSNE downscaling, clustering and visualization. The cell type of each cell cluster was then annotated using the SingleR package. The degree of malignancy of the hepatocytes in the data was assessed using the CytoTRACE algorithm and different cell subgroups were scored, with higher scores being associated with a higher degree of malignancy. Finally, the Monocle package was used to analyze the differentiation trajectories of hepatocellular carcinoma cells, and the CellChat package was used to analyze cell-to-cell interactions and receptor-ligand pairs between various cell types.

### 2.7 Risk score and treatment sensitivity

Chemotherapeutic drug and drug pathway information files were obtained from the Genomics of Drug Sensitivity in Cancer (GDSC; https://www.cancerrxgene.org/) database. Oncopredict was used to predict the degree of response of chemotherapeutic drugs to high and low-risk groups. The correlation between Risk Score and 198 chemotherapeutic drugs was analyzed using FDR <0.01 and | Rs | > 0.3 as thresholds, and the targeted pathways of action of these drugs were analyzed. The Immunophenotype Score (IPS) and Tumor Immune Dysfunction and Rejection (TIDE), expression of the immune checkpoint PD-1, and Tumor Mutational Burden (TMB) were used to assess patient response to immunotherapy by analyzing these algorithms to assess whether the Risk score could be used for immunotherapy prognostic assessment.

### 2.8 Cell culture and RT-qPCR assays

Human normal liver cell L-O2 and human hepatocellular carcinoma cell Huh7 were awarded by the research group of Wulin Yang, Translational Medicine Center, Institute of Health and Medical Technology, Hefei Institute of Physical Science, Chinese Academy of Sciences. In this study, cells were cultured in 1,640 and DMEM medium (corning, USA) supplemented with 10% fetal bovine serum (FBS, EXCELL) and 5% streptomycin/penicillin (Hyclone). The culture environment was humid at a constant temperature of 37° C and 5% CO2. Total RNA was extracted using the RNA Easy Fast Tissue/Cell Kit (DP451,TIANGEN). First-Strand cDNA was synthesized using TransScript First-Strand cDNA Synthesis SuperMix (AE301-03, TransGen Biotech). Real-time PCR was performed using ChamQ Universal SYBR RT-qPCR Master Mix (Q711-02, Vazyme) in a X960 Automatic medical PCR analysis system (Lixin Instruments (Shanghai) Co., LTD.). Relative mRNA expression levels were calculated from threshold cycle (Ct) values for each PCR product and normalized by β-actin using the comparative 2-△△Ct method.Primers are listed in Supplementary Table S1.

### 2.9 Statistical analysis

All analyses and data visualization were performed using R (http://www.r-project.org, version 4.3.1) or GraphPad Prism software (GraphPad Software, Inc., version 9.0). Survival differences were compared using the log-rank test, the Kruskal–Wallis test was used to test the relationship of continuous variables between the three groups, and the Wilcoxon rank-sum test was used to compare the relationship between variables in the two groups. Correlations between variables were analyzed using Spearman or Pearson statistics, and *p* values < 0.05 were considered statistically significant.

## 3 Result

### 3.1 Determination of VM-related molecular subtypes

**FIGURE 1:**
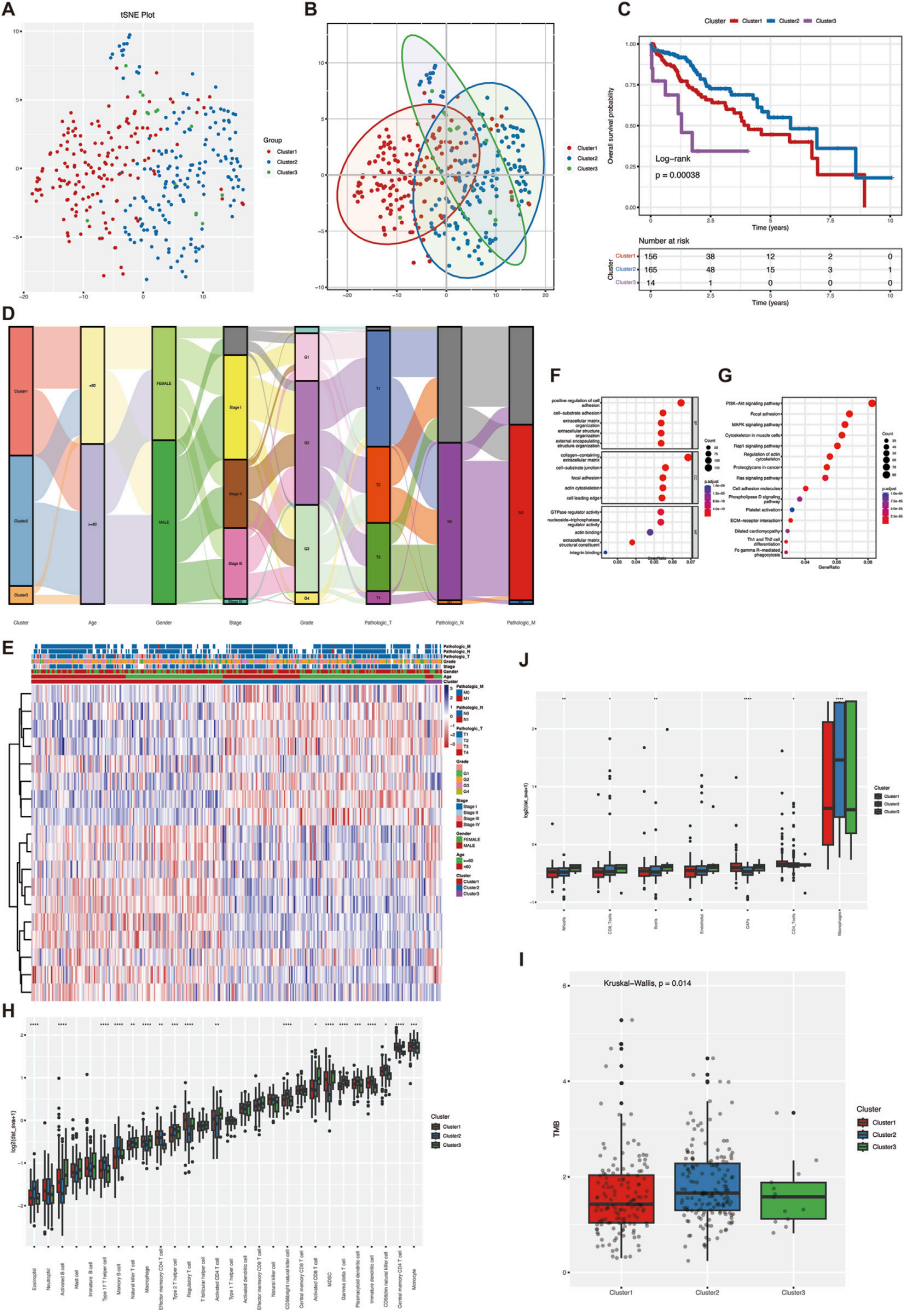
Molecular subtypes and heterogeneity related to vascular mimicry in hepatocellular carcinoma. **(A)** To ensure the stability of clustering, 1,000 times unsupervised consensus clustering method was used to classify the patients in the TCGA-LIHC cohort, and principal component analysis was used to reduce the scatter plot of the clustering **(B)** PCA analysis was performed on TCGA-LIHC data to distinguish between the three VM-related subgroups. **(C)** Kaplan-Meier curves for OS of the three VM clusters **(D)** Mulkey shunt plots showing changes in VM clusters, age, sex, grade, stage, and TNM stage. **(E)** heatmap of clinical features of differentially expressed genes in clusters **(F)** GO enrichment analysis of VM genes in TCGA cohort. **(G)** KEGG enrichment analysis of VM genes in TCGA cohort **(H)** landscape of immune cell infiltration among the three VM clusters. **(I)** TMB scores among the three VM clusters **(J)** epic scores among the three VM clusters. Statistical significance level ns ≥ 0.05, ∗< 0.05, ∗ ∗ ∗ < 0.01, ∗ ∗ ∗ < 0.001, ∗ ∗ ∗ ∗ < 0.0001.

Based on the expression levels of 18 VM-related genes, the optimal K value of three was determined based on the results of clustering consensus value and cumulative distribution function, and the TCGA-LIHC data were classified into three subtypes, namely, Cluster1 (n = 156), Cluster2 (n = 165), and Cluster3 (n = 14). The t-distribution random neighborhood embedding (t-SNE) algorithm showed that the three subtypes could be clearly distinguished (Figures 1A, B). Kaplan-Meier (K-M) survival analysis showed that the overall survival of Cluster two was significantly better than the other two groups (Figure 1C). Triage maps revealed significant differences in clinical features among the three subtypes (Figure 1D). The heat map reflected the expression of the three subtypes in pathological features, and the results revealed significant differences in tumor stage and pathological grade among the three subtypes (Figure 1E). The above results indicated that the subtypes were highly heterogeneous based on genes associated with angiogenesis. GO and KEGG enrichment analyses could determine whether the genes associated with VM have a unique transcriptome that promotes the growth of hepatocellular carcinoma (HCC) cells. GO and KEGG enrichment analysis identified 4,045 differentially expressed genes, which were shown to be significantly associated with cell proliferation pathways. Molecular function (MF) analysis showed that antigen binding and growth factor binding were associated with angiogenic genes (Figure 1F) and that these genes were enriched in the PI3K-Akt pathway (Figure 1G). This suggests that differential genes in VM subtypes play an important role in the proliferation of hepatocellular carcinoma.

Further comparing the heterogeneity of the tumor immune microenvironment among subtypes, ssGSEA results showed that in terms of the infiltration amount of “activated B-cells”, “type 17 helper T-cells” and “eosinophils” infiltration amount, there was a significant difference between the three groups (Figure 1H). In addition, the TMB score results showed that the TMB score of Cluster two was significantly higher than that of the other two groups (Figure 1I). The mutation types of the three groups were relatively consistent (Supplementary Figure S1), while the EPIC score showed that the overall proportion of immune cells was higher in Cluster2 than in the other two groups, whereas Cluster1 and Cluster3 had higher proportions of cancer-associated fibroblasts (CAF) (Figure 1J). Overall, the tumor purity of Cluster1 and Cluster3 was higher than that of Cluster2, and there was a significant difference in the degree of immune cell infiltration between the subtypes, which may be one of the reasons for the prognostic differences between the subtypes. The VM subtypes are a good indicator for evaluating the clinical prognosis of the patients and the heterogeneity of the TME.

### 3.2 Construction and validation of risk models associated with angiogenesis

**FIGURE 2:**
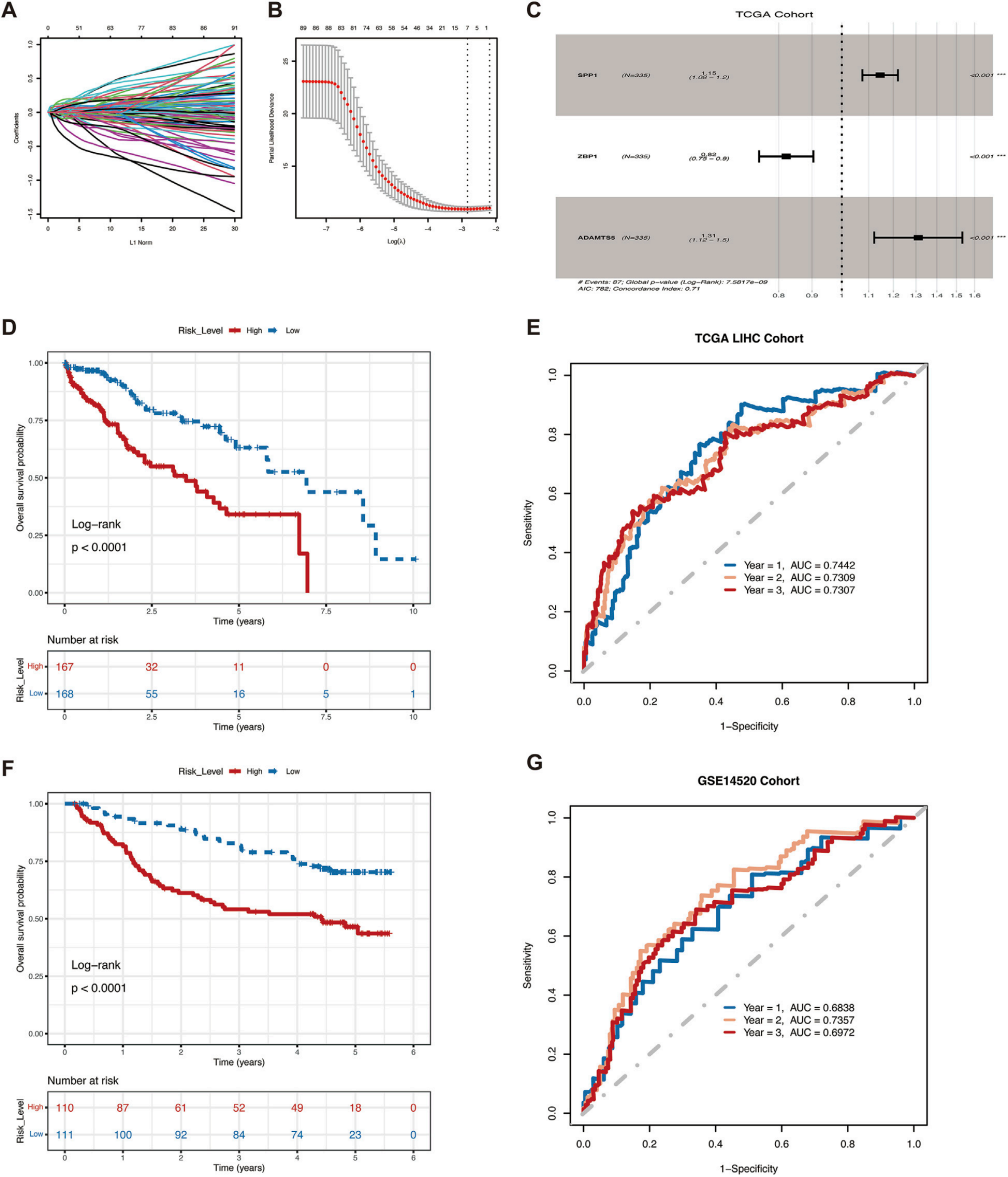
VM Score predict survival in HCC patients.**(A)** Ten-time cross-validation for tuning parameter selection in the LASSO model. **(B)** LASSO coefficient profiles of seven mRNAs. **(C)**Three differential genes used to construct the model. **(D)** Kaplan-Meier curves of OS for patients with high and low VM Score in the TCGA cohort. **(E)** ROC curves for the model training sets. **(F)** Kaplan-Meier curves of OS for patients with high and low VM Score in the GSE14520 **(G)** ROC curves for the model validation sets.

Survival analysis was used to screen 173 genes associated with survival from 4,045 differentially expressed genes in VM subtypes, and seven genes were further screened using the LASSO algorithm (Figures 2A, B), and the risk ratios (HR) of these genes were analyzed using multifactorial Cox regression. SPP1, ZBP1 and ADAMTS5 were finally selected for model construction (Figure 2C). Survival curves showed the association of these three genes with survival prognosis (Supplementary Figure S2). The risk score for each HCC patient could be calculated using the following formula:

VM Score = SPP1 * (0.07782104) + ZBP1 * (−0.15394666) + ADAMTS5 * (0.25495905).

To further assess the predictive ability of the prognostic model, we categorized patients into high-risk and low-risk groups based on the median risk score. Patients with low-risk scores in the training set showed significant survival benefits and tended to have a higher probability of survival than patients with high-risk score (Figure 2D). Predictive efficacy was assessed by the area under the ROC curve (AUC), which was 0.7442, 0.7309, and 0.7307 for 1, 2, and 3 years, respectively (Figure 2E). The robustness of this prognostic risk model was further validated on the GSE14520 dataset. Conistent with the training set, the survival curves similarly showed that the survival rate of the high-risk group was significantly lower than that of the low-risk group (Figure 2F), with AUC of 0.6838, 0.7357, and 0.6972 at 1, 2, and 3 years, respectively (Figure 2G). The above results demonstrated the good performance of VM Score in predicting the prognosis of HCC.

### 3.3 Construction and evaluation of nomogram models

**FIGURE 3:**
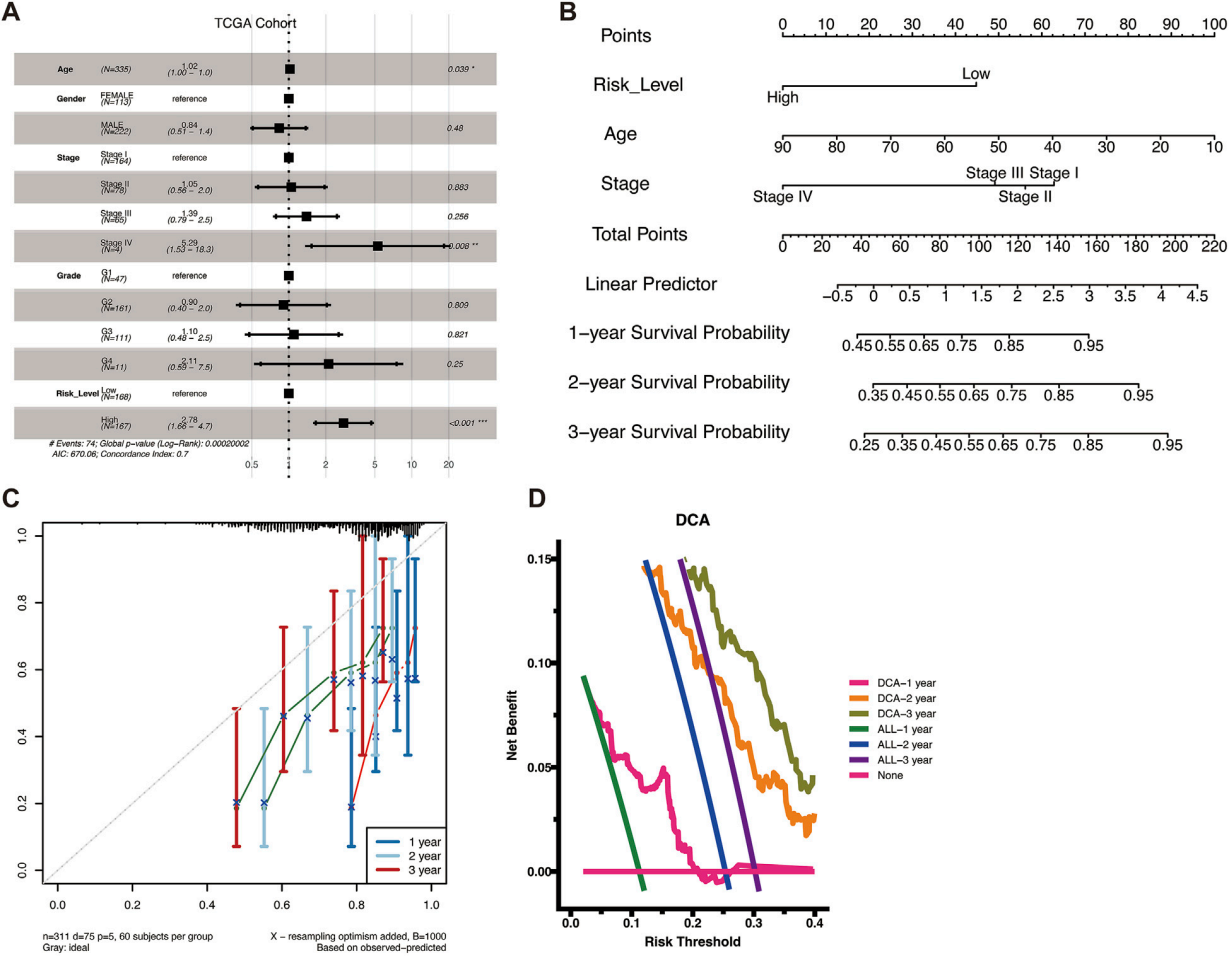
Construction of a nomogram. **(A)** Forest plots of univariate and multivariate analyses including risk scores and clinical factors in TCGA cohort. **(B)** Nomograms for predicting the 1–3-year survival probabilities of HCC patients in the TCGA dataset. **(C)** Calibration plot for predicting 1–3-year OS of HCC patients in the TCGA cohort. The survival probability predicted by the nomogram was plotted on the *X*-axis. Actual survival rates are plotted on the *Y*-axis. **(D)** DCA curve to evaluate the clinical validity of the prediction model.

To verify whether VM Score was an independent prognostic factor for HCC patients, we further performed a multivariate Cox regression analysis of clinical characteristics and VM Score in the TCGA-LIHC patient dataset. The results showed that the risk ratio (HR) for high VM Score was 2.78, demonstrating that VM Score was an independent prognostic factor for HCC patients (Figure 3A). Combining clinical information and VM Score, we developed a Nomogram model that can be used clinically to predict the survival of HCC patients from 1 to 3 years (Figure 3B). By summing the scores for each prognostic parameter, a total score can be obtained for each patient, with higher total scores indicating poorer patient survival. The calibration curve showed that the predicted survival probability was in good agreement with the actual survival probability, validating the reliability of the Nomogram model (Figure 3C). Decision curve analysis (DCA) showed that the prognostic values of the column-line graph were better than those of the individual variables (Figure 3D). In conclusion, VM Score can be used as a reliable method to predict the survival of HCC patients in clinical practice.

### 3.4 VM Score risk factor analysis in single-cell data

**FIGURE 4:**
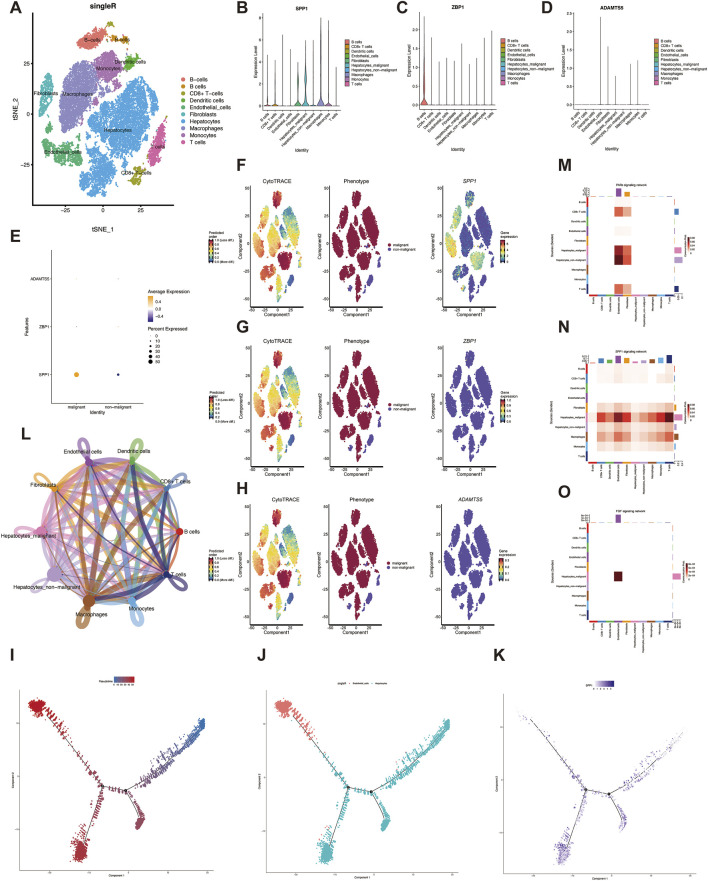
Single cell analysis. **(A)** Identification of cell types by marker genes. **(B–D)** expression of three genes in eight cell types. **(E)** Expression of three genes in malignant and non-malignant cells. **(F–H)** The expression of three genes during the development of liver malignant cells. **(I–J)** Pseudo-time series analysis of different cell types during the development of hepatic malignant cells **(K)** Expression of SPP1 at different time nodes during the development of liver malignant cells. **(L)** The number of interactions in the intercellular communication network. **(M)** Different cellular interactions in the PARs signaling pathway. **(N)** cell-cell communication interactions in the SPP1 signaling pathway. **(O)** The interaction between hepatocytes and endothelial cells was significant in the FGF pathway.

Single-cell sequencing enables analysis of tumor heterogeneity and reveals properties of the tumor microenvironment at single-cell resolution. We first performed integration analysis on the GSE149614 dataset and removed sample batch differences based on the mean and dispersion of SPP1, ZBP1 and ADAMTS5. Then, after performing PCA downscaling, the first 20 dimensions were retained, and the clustering results showed that 31,286 cells were annotated into nine different cell types for subsequent analysis (Figure 4A). To further characterize the proportion of angiogenic VM score in tumor cells, we analyzed the expression of these three genes in different cells (Figures 4B–D). SPP1 was increased in hepatic malignant cells, while CD8^+^ T cells were also increased, suggesting that SPP1 may play an important role during HCC invasion and metastasis. We analyzed the expression of genes in VM Score in malignant cells, and the results showed that SPP1 expression was significantly elevated in hepatic malignant cells (Figure 4E).

To elucidate the degree of malignancy of the cells in the HCC single-cell sequencing data, we found that different subpopulations of hepatocytes with higher differentiation potential could be transformed into hepatic malignant cells by CytoTRACE scoring. In addition, SPP1 was highly expressed in hepatic malignant cells, which may indicate that SPP1 expression is positively correlated with hepatic malignant cells (Figure 4F), whereas ZBP1 and ADAMTS5 were not significantly correlated with hepatic malignant cells (Figures 4G, H). To characterize the temporal status of vascular endothelial cells during HCC development, we used Monocle to rearrange individual cells into a pseudo-timeline, and the results demonstrated the homogeneous progression of endothelial cells to hepatic malignant cells (Figures 4I, J), and showed the SPP1 expression at different developmental nodes of hepatocytes (Figure 4K). Understanding the interactions between tumor cells and immune cells helps to further understand the potential causes of cancer progression and metastasis, so we explored the intercellular communication network by calculating the interaction probabilities, and the results showed stronger interactions between tumor cells and other immune cells (Figure 4L). In addition, cellular communication networks were hypothesized based on specific signaling pathways and ligand-receptor interactions, and malignant and endothelial cells were found to be closely associated with the communication networks of PARs, SPP1, and FGF signaling pathways (Figures 4M–O). These pathways may create conditions for the malignant proliferation of tumor cells in HCC, further revealing the role of VM Score in the tumor microenvironment.

### 3.5 Potential therapeutic effects of VM score

**FIGURE 5:**
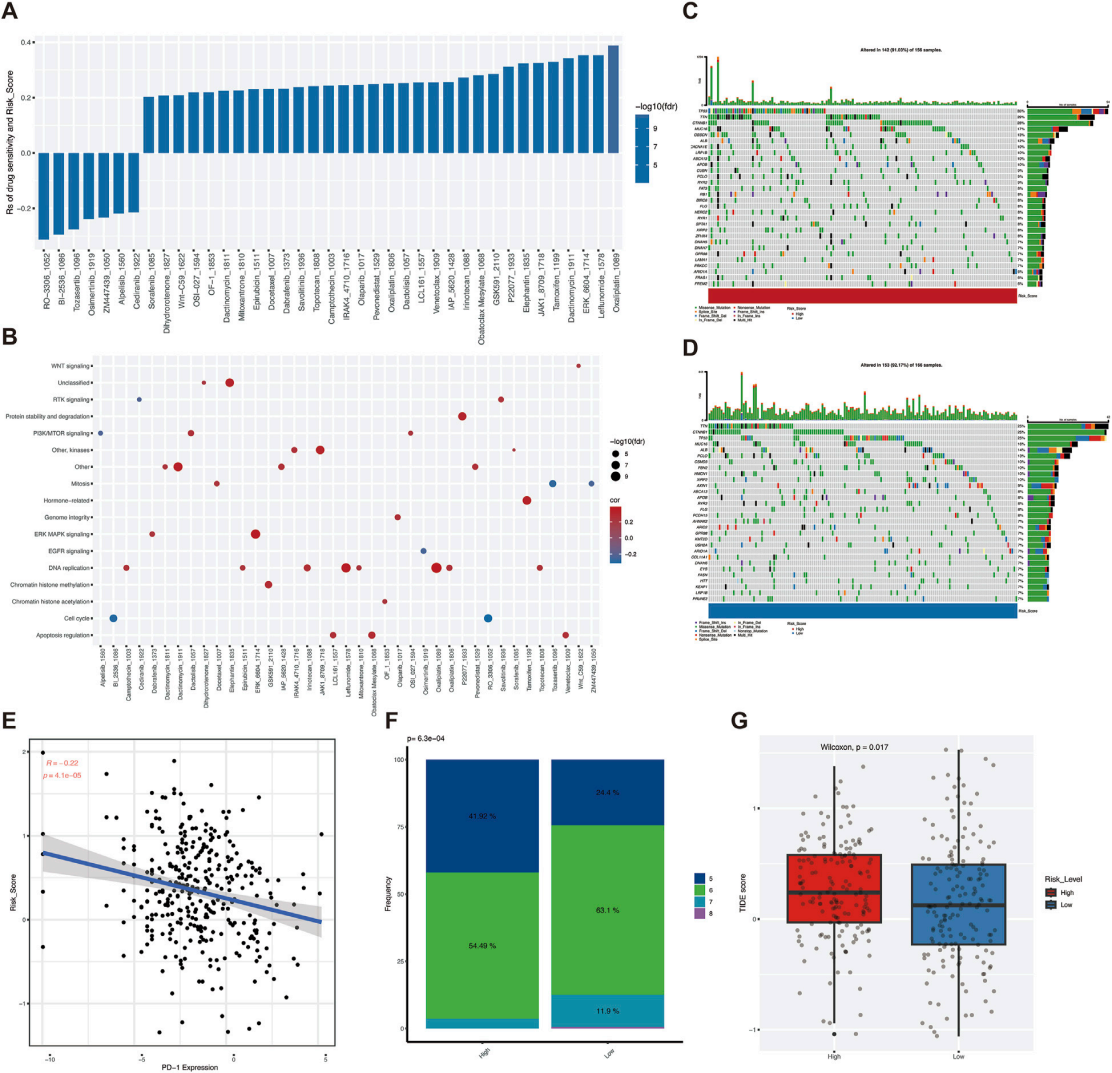
VM score-related HCC immunotherapy and drug therapy. **(A)** Of the 39 drugs significantly associated with risk scores, seven had a sensitive association with VM Score. **(B)** pathway analysis of drug targeting. **(C)** Waterfall plots showing the mutation distribution of the top 30 most frequently mutated genes in the high risk groups. **(D)** Waterfall plots showing the mutation distribution of the top 30 most frequently mutated genes in the low risk groups. **(E)** Correlation between Expression of immune checkpoint molecules PD-1 and VM Score. **(F)**IPS scores of high - and low-risk groups. **(G)**TIDE scores of high - and low-risk groups.

Previous results suggest that VM Score has clinical applications. To further evaluate whether VM Score can be used as a biomarker for clinical treatment, we analyzed the association between VM Score and the response of HCC patients to chemotherapeutic agents and immunotherapy. First, we evaluated the IC50 values of VM Score for different chemotherapeutic agents, and the drug sensitivity results showed that the sensitivities of seven drugs, including a small molecule inhibitor targeting PLK1 (BI-2536–1,086) and a selective inhibitor of CDK1 (RO-3306–1,052), were positively correlated with VM Score (Figure 5A). Next, we explored the signaling pathways targeted by the drugs, and we found that the drugs whose sensitivities were positively correlated with the VM Score were mainly targeted in the cell cycle pathway (Figure 5B). Taken together, VM Score may be a potential biomarker for developing appropriate drug treatment strategies.

Immunotherapy is a promising clinical treatment for tumors, and it is particularly important to find a biomarker that can predict the prognosis of immunotherapy; we analyzed the correlation between immunotherapy prognostic markers and VM Score. The waterfall plot demonstrated the mutation differences in the first 30 genes between high and low VM Score, with a DNA mutation frequency of 91.03% in the high-risk group (Figure 5C) and 92.17% in the low-risk group (Figure 5D). The mutation frequency was higher in the low-risk group. Correlation analysis based on the expression of immune checkpoint molecules showed a stronger clinical response in the low-risk group (Figure 5E). The results of the IPS score suggested that HCC patients in the low-risk group might benefit from immunotherapy (Figure 5F). To assess the responsiveness of patients in different risk groups to immunotherapy, the TIDE score was used for prediction, which was significantly lower in the low-risk group (Figure 5G). In conclusion, the VM Score provides new ideas for immunotherapy regimens and chemotherapeutic drug application in HCC, and patients in the low-risk group may have a higher response rate to immunotherapy.

### 3.6 RT-qPCR to verify the expression of angiogenesis-related genes

**FIGURE 6:**
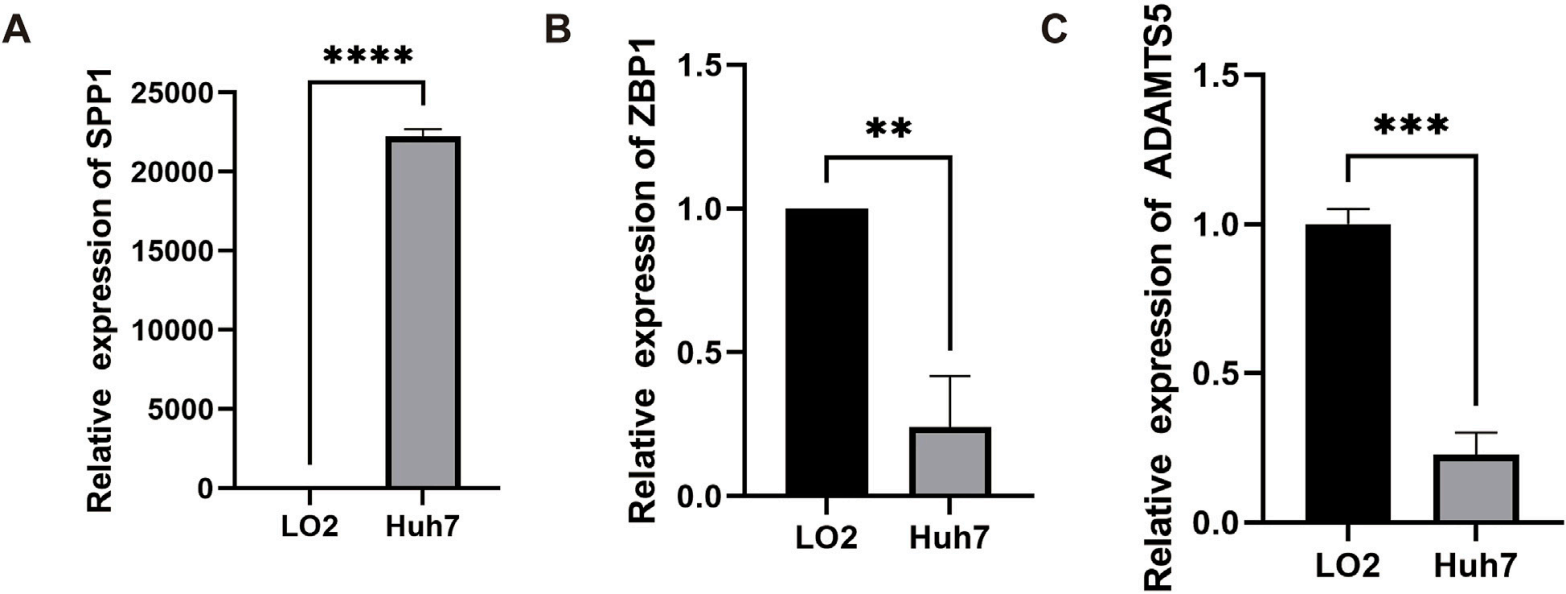
Gene expression validation in HCC cell lines. Histograms of different transcript levels of SPP1 **(A)**, ZBP1 **(B)** and ADAMTS5 **(C)** in Huh7 and LO2.

Finally, to understand the expression of the three genes in VM Score, the expression levels of these three genes in HCC cells and human normal liver epithelial cells were verified by quantitative real-time PCR (RT-qPCR). The results showed that the SPP1 mRNA level in HCC cells Huh7 was significantly higher than that in human normal liver epithelial cells LO2 (Figure 6A), whereas the expression of ZBP1 and ADAMTS5 was lower than that of LO2 in Huh7 (Figures 6B, C), with a significant difference of *p*-value < 0.05.

## 4 Discussion

Liver cancer is one of the most common malignant tumors in humans, with hepatocellular carcinoma (HCC) being the most common type of liver cancer. The incidence of HCC is increasing due to the current epidemics of viral hepatitis, alcoholism, and nonalcoholic steatohepatitis, and HCC has attracted widespread attention worldwide (Huang et al., 2021; Sung et al., 2021; Ilagan-Ying et al., 2024). Currently, the main therapeutic approaches for HCC include hepatectomy, liver transplantation, monoclonal antibodies, and molecularly targeted therapy with small molecule compounds (Rinaldi et al., 2021). HCC is a highly immunogenic malignant tumor surrounded by a large number of immune cells. TME significantly influences the development and progression of HCC, and the research on TME-based immunotherapeutic response has progressed and gained recognition by hepatocellular carcinoma patients worldwide (Liu et al., 2019). Despite some advances in HCC treatment, the rapid progression and metastasis of advanced HCC leads to poorer prognosis and higher mortality (Llovet et al., 2021), which requires the development of reliable prognostic biomarkers to improve the survival of patients with advanced HCC. VM has been identified as a microcirculatory pattern that promotes tumor angiogenesis and may be a target for antitumor therapy (Zhao et al., 2015). To better apply this feature of VM to the clinical evaluation of HCC patients, we typed the samples by VM and constructed a prognostic model of HCC consisting of ZBP1, SPP1, and ADAMTS5 by using the characteristic genes of the subtypes, which achieved good predictive efficacy and provided a new idea for drug therapy and immunotherapy.

ZBP1 (Z-DNA-binding protein 1) is a protein that can be induced by IFN (Yang et al., 2021). ZBP1 acts as a necroptotic apoptotic signaling pathway that activates inflammatory cell death. Previous studies have shown that ADAR1 inhibits ZBP1-mediated apoptosis in inflammatory cells, thereby promoting tumorigenesis (Yang et al., 2020). Yang et al. showed that restoration of ZBP1 expression in the human colon cancer cell line HT-29 promotes the inhibitory effect of chemotherapy on tumor growth (Yang et al., 2023). These results suggest that ZBP1 plays an important role in tumor development. In this study, we found that ZBP1 expression was associated with a better prognosis, and experiments showed that the expression of ZBP1 was significantly higher in normal cells than in tumor cells.

Secreted phosphoprotein 1 (SPP1, also known as osteoblast protein, OPN) is an extracellular matrix protein, and previous studies demonstrated the prognostic value of SPP1 and the potential role of HCC-secreted SPP1 in TME of HCC patients (Liu et al., 2022). In our study, we demonstrated the high expression of SPP1 in HCC cells, the higher percentage of SPP1 in malignant cells, and a poorer prognosis.

The ADAMTS (integrin and metalloproteinase containing platelet adhesion structural domains) family includes 19 secreted zinc metalloproteinases that have been found to play a role in a variety of biological and pathological processes (Kelwick et al., 2015). In this study, ADAMTS5 was significantly downregulated in HCC cell lines. However, our bioinformatics analysis showed that high expression of ADAMTS5 was associated with poorer prognosis in HCC patients, which needs to be further verified in more samples.

In hepatocellular carcinoma, the interactions among SPP1, ADAMTS5 and ZBP1 are complex and may synergize in multiple mechanisms.ADAMTS5 and SPP1 may interact in regulating immune cell infiltration in the tumor microenvironment, and ADAMTS5, by degrading the ECM (Mead and Apte, 2018), may enhance the role of SPP1 in the tumor microenvironment.ZBP1, by inducing necrotic apoptosis, may promote local inflammatory responses within tumors (Zheng and Kanneganti, 2020), which together with SPP1 and ADAMTS5 drive tumor progression.

In a previous study, LOXL2, a gene that promotes VM development and is involved in the regulation of TME, has been validated as a potential diagnostic and prognostic biomarker as well as a therapeutic target for HCC, suggesting that VM-associated genes have the potential to provide new targets and strategies for the treatment of hepatocellular carcinoma (Zhao et al., 2023).

The emergence of VM is often accompanied by the accumulation of immunosuppressive cells, such as myeloid-derived suppressor cells (MDSCs), in the tumor microenvironment (Li et al., 2021). These immunosuppressive cells weaken the immune system’s recognition and clearance of tumors by inhibiting the function of effector immune cells. Therefore, the immunosuppressive microenvironment established in hepatocellular carcinoma through VM may lead to resistance to immunotherapy. The formation of VM is often accompanied by localized hypoxia within the tumor, and this hypoxic environment has a significant impact on chemotherapeutic agents, thereby reducing their effectiveness (Haiaty et al., 2020). By correlation analysis with chemotherapeutic and immunotherapeutic drugs, VM Score may serve as an effective HCC biomarker for predicting patient response to chemotherapeutic drugs and immunotherapy.

Tumor mutational load (TMB) has been proposed as a predictive biomarker of immune response to tumors, and the accumulation of somatic mutations is one of the major causes of tumorigenesis (Gubin et al., 2015). We further analyzed TMB in the high and low-risk groups and found that the mutation frequency was higher in the low VM Score group, which is consistent with our previous results in the immune checkpoint molecular expression analysis. Interestingly, TTN, TP53, and CTNNB1 were the genes with the highest mutation frequencies in both the high- and low-risk groups, but the mutation frequencies of these three genes were higher in the high-risk group than in the low-risk group.TP53 mutation is one of the most common mutations in HCC and affects the progression and prognosis of HCC (Long et al., 2019), and TTN and TP53 mutations often occur in HCC samples simultaneously and also mediate the prognosis of HCC patients (Gao et al., 2021).CTNNB1 mutation is associated with ALDOA phosphorylation and was verified to promote glycolysis and cell proliferation (Gao et al., 2019), which may explain the higher purity and rapid proliferation of malignancies in the high-risk group.

Our further analysis of single-cell data revealed that VM influences the tumor microenvironment in HCC that SPP1 positively correlates with the degree of hepatocellular malignancy during hepatocellular malignant transformation, and that endothelial cells, as a part of the tumor microenvironment, are involved in angiogenic signaling along with pro-angiogenic factors secreted by tumor cells (De Visser and Joyce, 2023). CellChat analysis showed that hepatic malignant cells have strong interactions with endothelial cells in PARs, FGF, and SPP1 signaling pathways; the receptor-ligand model of hepatic malignant cells and endothelial cells provides a rationale for new immunotherapeutic targets.

In HCC patients, VM is closely associated with TME, and targeting VM can modulate immune cell infiltration and function, providing a new strategy for immunotherapy. Although the implementation of VM as a target for immunotherapy is full of potential, it still faces many challenges in clinical practice. Firstly, tumors have immune escape mechanisms, and targeting VM alone may not be sufficient to overcome all immune escape mechanisms, resulting in limited therapeutic efficacy, which may require us to combine immune checkpoint inhibitors. Secondly, there is limited data in clinical trials and more preclinical validation is needed subsequently.

This study aimed to classify HCC samples based on VM characteristics, construct a survival-related prognostic model, and provide personalized therapeutic recommendations for HCC patients. Although these studies revealed some associations between VM and HCC, there are some limitations of this study. First, all bioinformatics data were obtained from public databases, and some samples lacked detailed clinical information. The data were derived from retrospective studies, and the sample size was insufficient to fully cover the characteristics of the entire HCC patient population. Second, although our RT-qPCR validation confirmed the mRNA expression of genes in HCC cell lines, the results of the immune infiltration and other analyses were derived from bioinformatic analyses and lacked basic experimental validation, which may lack reliability.

## 5 Conclusion

In any case, our study shows that VM related genes play a role in promoting tumor cell proliferation in HCC by affecting TME. The VM Score constructed by VM related genes has prognostic characteristics and is closely related to tumor immunotherapy and chemotherapy.

## Data Availability

The original contributions presented in the study are included in the article/Supplementary Material, further inquiries can be directed to the corresponding authors.

## Abbreviations

GTEx, Genotype-Tissue Expression; GEO, comprehensive gene expression database; TCGA, The Cancer Genome Atlas; PCA, principal component analysis; ssGSEA, single-sample gene set enrichment analysis; AUC, area under the receiver operating curve; ROC, Receiver operating characteristic; TMB, tumor mutation burden; CNV, Copy number variation; HCC, Hepatocellular carcinoma; TME, Tumor microenvironment; OS, Overall survival; KEGG, Kyoto Encyclopedia of Genes and Genomes; GO, Gene Ontology; LASSO, Least absolute shrinkage and selection operator; VM, Vasculogenic mimicry; DCA, Decision Curve Analysis; EPIC, Estimating the Proportion of Immune and Cancer cells; RT-qPCR, Real-time Quantitative Reverse Transcription Polymerase Chain Reaction; TCIA, The Cancer Immunome Atlas; GDSC, Genomics of Drug Sensitivity in Cancer.
