# Supplementary material for: A comprehensive analysis of vasculogenic mimicry related genes to predict the survival rate of HCC and its influence on the tumor microenvironment
Source: Front Genet. 2024 Dec 19;15:1437715. doi: 10.3389/fgene.2024.1437715 (PMC11693674; doi:10.3389/fgene.2024.1437715)
Supplement: Supplementary file 1 [file Table1.docx]

Additional file 2

**This file includes:**

Figure.S1-S2


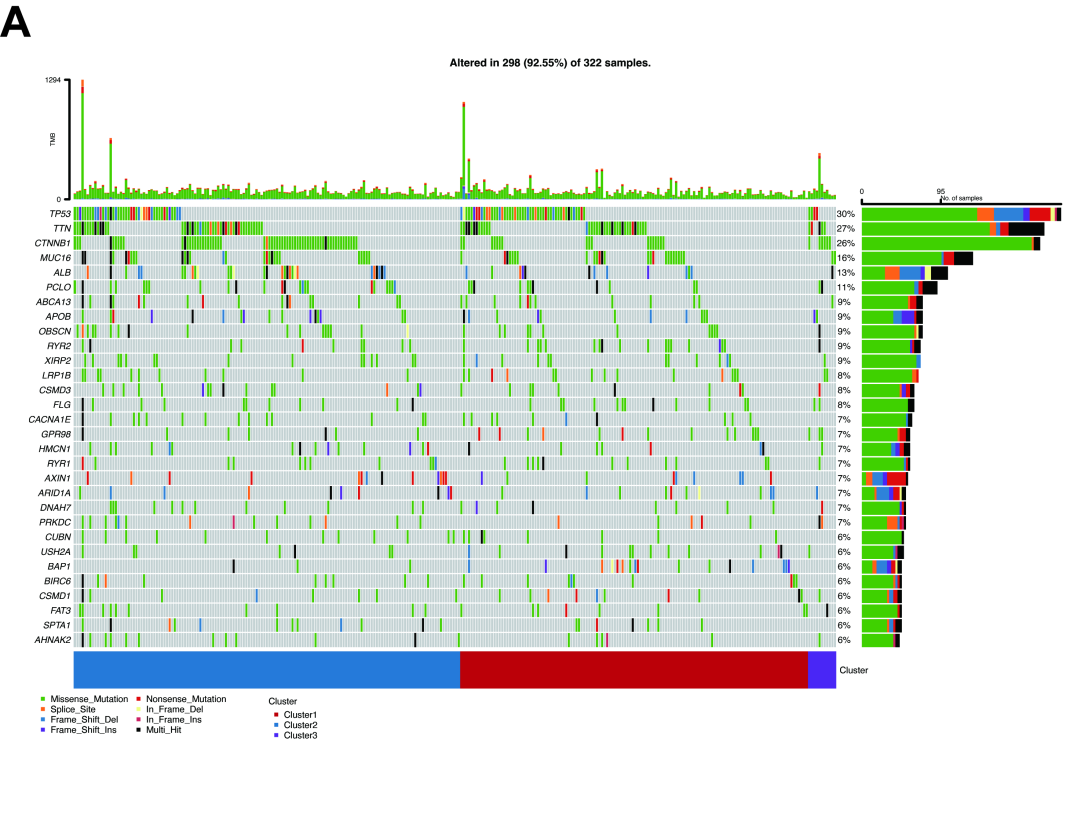


**Additional file 2: Figure S1.**

1. Waterfall map of mutation of three vm clusters.


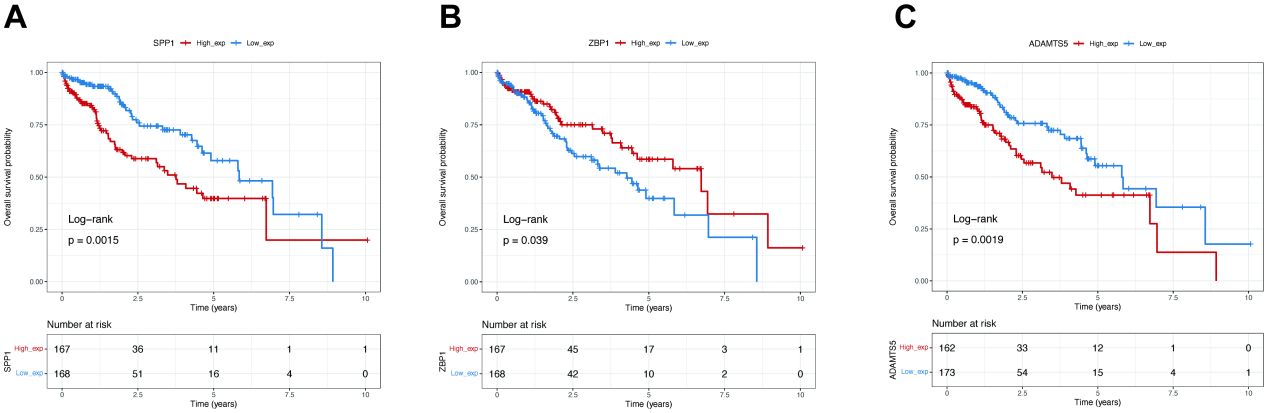


**Additional file 2: Figure S2.**

（A）Kaplan-Meier curves of OS of SPP1 in TCGA-LIHC cohort.（B）Kaplan-Meier curves of OS of ZBP1 in TCGA-LIHC cohort.（C）Kaplan-Meier curves of OS of ADAMTS5 in TCGA-LIHC cohort.
