# Supplementary material for: A comprehensive analysis of vasculogenic mimicry related genes to predict the survival rate of HCC and its influence on the tumor microenvironment
Source: Front Genet. 2024 Dec 19;15:1437715. doi: 10.3389/fgene.2024.1437715 (PMC11693674; doi:10.3389/fgene.2024.1437715)
Supplement: Supplementary file 2 [file Table2.docx]

**Additional file 1: Table S1. Primer design sequence for q-PCR.**

| Gene | Forward primer (5’-3’ on plus strand) | Reverse primer (5’-3’ on plus strand) |
| --- | --- | --- |
| β-actin | CATCCGCAAAGACCTGTACG | CCTGCTTGCTGATCCACATC |
| SPP1 | GACCCATCTCAGAAGCAGAATCTC | CGTGGGAAAATCAGTGACCAGTTC |
| ADAMTS5 | GCTATCCTGTTTACTCGGGAGGAT | GTGGTAGGTCCAGCAAACAGTTAC |
| ZBP1 | CAACAACGGGAGGAAGACATCTAC | CATGCTTTGGACTGCTCATCCATG |
